# Nutritional and Antioxidant Potential of Fiddleheads from European Ferns

**DOI:** 10.3390/foods10020460

**Published:** 2021-02-19

**Authors:** Marcela Dvorakova, Karolína Pumprova, Žaneta Antonínová, Jan Rezek, Daniel Haisel, Libor Ekrt, Tomas Vanek, Lenka Langhansova

**Affiliations:** 1 The Czech Academy of Sciences, Institute of Experimental Botany, Rozvojova 263, CZ-16502 Prague, Czech Republic; dvorakova@ueb.cas.cz (M.D.); karolina@herbafabrica.com (K.P.); antoninova.zaneta@seznam.cz (Ž.A.); rezek@ueb.cas.cz (J.R.); haisel@ueb.cas.cz (D.H.); vanek@ueb.cas.cz (T.V.); 2 Faculty of Agrobiology, Food and Natural Resources, Czech University of Life Sciences Prague, Kamýcká 129, CZ-16521 Prague, Czech Republic; 3 Faculty of Science, University of South Bohemia, Branisovska 1760, CZ-37005 Ceske Budejovice, Czech Republic; libor.ekrt@gmail.com

**Keywords:** fern species, fiddleheads, fatty acid profile, natural antioxidants, nutritional quality, alternative vegetable

## Abstract

Ferns are part of the diet and traditional medicine in East Asia, North America, and Oceania, however, their importance has been forgotten in Europe. Here, the nutritional and antioxidant potential of young fern fronds (fiddleheads) of eight families were studied. Most of the tested fern species excelled in high antioxidant capacity when compared to the reference leafy vegetables spinach and rocket. On average, the total phenol content reached 220 mg·g^−1^ of extract dry weight for all fiddleheads, and 15 out of 24 tested species exceeded 1 g Trolox equivalent per gram of extract dry weight in Oxygen Radical Absorbance Capacity (ORAC) assay. On the other hand, fiddleheads contained a comparable amount of carotenoids and ascorbic acid with the reference vegetables. In the case of fatty acid composition, fiddleheads contained especially high amounts of essential omega-3 (n3) and omega-6 (n6) polyunsaturated fatty acids with a beneficial n6/n3 ratio. The n6/n3 ratio in all tested species was between 2 and 6.4, whereas the ratio in the reference vegetables was below 0.4. All in all, fiddleheads from European ferns are a rich source of valuable antioxidants and essential fatty acids with a desirable n-6/n-3 ratio and may thus form an alternative source of these compounds, especially for those people not consuming fish and fish products.

## 1. Introduction

Ferns have been recognized as edible medicinal plants for centuries, especially in China, India, and other Asian countries. Taxonomically, they belong to pteridophytes, vascular plants that disperse via spores [1]. Ferns (*Monilophyta*) are represented by about 12,000 species [2]. The habit of eating ferns goes back thousands of years when wild plant gathering was a primary food source. For example, edible ferns are mentioned in Chinese literature as early as 3000 years ago [3]. In addition, the use of fern extracts has already been known in ancient medicinal systems of India (Ayurveda) and China [3]. Although today, wild plant gathering is not as widespread as thousands of years ago, there are still regions where fern harvesting has remained routine or is even used commercially, such as New Zealand [4], Japan, and the USA [5], Canada [6], China, Nepal, India, Malaysia, and the Philippines [3]. It may be said that it is even on the rise, as more and more people are seeking healthier lifestyles and a return to traditional customs. Ferns, as plants with an established impact on human health, have thus regained attention. Extracts of fern parts has been studied with the aim to establish their biological activity as well as to identify the responsible chemical entities. Fern extracts exhibited potent antioxidant, antimicrobial, antibacterial, antiviral, and anti-inflammatory activities [7,8]. Simultaneously, ferns have been found to contain a high amount of phenolic compounds, glycosides, flavonoids, terpenoids, carotenoids, alkaloids, and fatty acids [7,9]. Their consumption is thus valuable not only from the medicinal point of view, but also from a nutritional perspective. When compared to other green vegetables, ferns are especially rich sources of antioxidants and of essential omega-3 and omega-6 fatty acids [10,11]. On the other hand, there is a concern about the potential toxicity of fern species, which will be discussed further.

Herein, the nutritional value of 24 mainly European fern species belonging to eight families was determined in fern fiddleheads (young fronds). The total content of carotenoids, phenolic compounds, fatty acids, and ascorbate was measured along with the identification and quantification of individual carotenoids and fatty acids. The antioxidant capacity was also evaluated. For the comparison of the obtained values, two green leafy vegetables, spinach, and rocket, were selected as reference vegetables as their young leaves are commonly consumed and sold in supermarkets.

## 2. Materials and Methods

### 2.1. Plant Material and Chemicals

The young fern fronds (fiddleheads) were collected (24 species, 8 families) during the spring period in 2018. All tested species were of European origin, cultivated for horticultural (bred cultivars) or botanical purposes (native species), or grown in the wild (supplementary Table S1). Preferentially we choose bred species (either native or horticultural) for our study since several species are endangered in Europe. However, neither bred nor native cultivars grown in botanical gardens were available for *Preridium aquilinum*. Thus, it was collected in the wild as it belongs to one of the most common edible ferns. Few fern species were collected in duplicate—horticultural and botanical garden samples such as *Polystichum aculeatum*, *Osmunda regalis*, and *Polypodium vulgare*; or—botanical garden and wild collected samples, such as *Lastrea limbosperma,* in order to detect possible variations caused by cultivation conditions (pot vs. soil, or soil vs. wild). Determination of studied taxa was provided by RNDr. Libor Ekrt, Ph.D. or by the authorities in the Botanical Garden of the Charles University in Prague, Faculty of Sciences. The collected fiddleheads were kept on dry ice during the transport and then stored under −80 °C until extraction for particular analyses.

Reference vegetables, rocket (*Eruca sativa*) and spinach (*Spinacia oleracea*), were seeded from seeds and grown in the pots in the greenhouse for two months until young leaf collection.

All chemicals and reagents for analyses (Folin-Ciocalteu reagent, fluorescein, 2,2’-azobis(2-methylpropionamidine) dihydrochloride) were purchased from Sigma-Aldrich s.r.o. (Prague, Czech Republic). Similarly, the organic solvents and standards (Trolox, gallic acid, L-ascorbic acid, nonadecanoic acid, methyl heptadecanoate-d_33_, Supelco 37 component FAME Mix) were purchased from Sigma-Aldrich s.r.o. (Prague, Czech Republic).

### 2.2. Total Phenol Content

The fiddleheads were freeze-dried using a FreeZone 2.5 Labconco Freeze-Dryer (Labconco corp., Kansas City, Missouri, USA) equipped with a vacuum pump (Vacuubrand GMBH + CO KG, Wertheim, Germany) at −50 °C, 0.370 mBar. Freeze-dried and homogenized (using a mortar and pestle) fiddleheads (0.5 g) were extracted twice in 10 mL of methanol overnight. The extraction was supported by sonication for 30 min in an ultrasound bath at room temperature in the dark. Combined extracts were centrifuged at 1730× *g*, 15 min (Centrifuge Hettich Universal 32R, Tuttlingen, Germany), at room temperature and then filtered through 30 mm polyvinylidene fluoride (PVDF) 0.45 µm membrane filter (ProFill, Fisher, Pardubice, Czech Republic). Filtrates were evaporated in a vacuum on a rotary evaporator at water aspirator pressure until almost dry, and then dried completely under nitrogen flow. The average extract yield was 26%. Obtained residues were redissolved in dimethyl sulfoxide (DMSO) to reach the concentration of 100 mg.mL^−1^ and the DMSO aliquots were kept at −80 °C for further analyses.

Total phenol content was determined in methanolic extracts by a modified Folin-Ciocalteu colorimetric method [12]. Briefly, the DMSO aliquots were diluted in water to reach the final concentration of 80, 60, and 40 µg.mL^−1^ in the reaction. The samples were then incubated with Folin-Ciocalteu reagent in 96-well flat-bottom transparent microplates, and after 10 min of shaking (200 rpm) at room temperature, the reaction was terminated using 12% anhydrous sodium carbonate. The absorbance was read at 760 nm after 30 min incubation in the dark at 37 °C. The calculation of the phenolic content was based on a calibration curve obtained for a reference compound, gallic acid, in a concentration range from 0.39 to 25 µg·mL^−1^. The total phenol content was expressed in milligrams of gallic acid equivalent per gram of dry extract (mg GA_eqv_·g^−1^ DW).

### 2.3. Evaluation of Antioxidant Capacity

The antioxidant capacity was measured using ORAC assay (Oxygen Radical Absorbance Capacity) according to Silva et al. [13]. Briefly, the DMSO aliquots (prepared as described above) were diluted in 75 mM potassium phosphate buffer (pH 7.0) to reach the final concentration of 10, 5, or 2.5 µg·mL^−1^, respectively, in the reaction. Each sample was measured at four serial concentrations obtained by 1:1 dilutions. The standard Trolox was prepared in six serial concentrations with the highest concentration 12.5 µg·mL^−1^. The sample or Trolox was incubated with 48 nM fluorescein for 30 min at 37 °C, and the reaction was started with 153 mM 2,2′-azobis(2-methylpropionamidine). The reaction kinetics were measured at 485 nm excitation and 535 nm emission wavelength for 1 h at 1 min intervals. The antioxidant capacity was calculated according to Cao and Prior [14] and expressed as milligrams of Trolox equivalent per gram of dry extract (mg T_eqv_·g^−1^ DW).

### 2.4. Determination of Ascorbate Content

About 60–80 mg of sample was homogenized in 2 mL of cooled 1.5% metaphosphoric acid and centrifuged at 1730× *g* for 5 min at 4 °C.

Separation was carried out on an isocratic HPLC system (ECOM, Prague, Czech Republic) consisting of Beta 10 pump, Sapphire detector (detection at 248 nm), HTA300 autosampler, Watrex Nucleosil column (120-5-C18, 250 × 4 mm, 5 µm particle size), and Clarity software (DataApex, Prague, Czech Republic). The solvent system was the mixture of 1 mM hexadecyltrimethylammonium bromide in 0.1 M acetic acid, the run time was 15 min, and the flow rate 1 mL·min^−1^. Calibration was done with commercially available L-ascorbic acid (reagent grade, crystalline, Sigma-Aldrich s.r.o., Prague, Czech Republic) using 5 different concentrations in range 5–200 µg·mL^−1^. The ascorbate content was expressed in micrograms per gram of fresh leaves (µg·g^−1^ FW).

### 2.5. Quantification of Carotenoids and Xanthophylls

Carotenoids were extracted from 5 mg of freeze-dried fiddlehead samples with 1 mL of acetone with 0.001% of an antioxidant, butylated hydroxytoluene (BHT, 2,6-di-*tert*-butyl-4-methylphenol, Sigma-Aldrich s.r.o., Prague, Czech Republic) and centrifuged for 5 min at 9000× *g* (microcentrifuge Sigma 1-14, Germany). The supernatant was separated, sediment was re-extracted and centrifuged as above, and both supernatants were then combined and evaporated under nitrogen flow. Dry pigment mixtures were stored at −80 °C and dissolved in 200 µL of acetone before analyses. The presence of carotenoids, such as neoxanthin, violaxanthin, antheraxanthin, lutein, zeaxanthin, and *β*-carotene, was detected using HPLC/UV-VIS system, consisting of a Gradient Pump Beta, Autosampler HTA 300; Watrex Nucleosil column (120-5-C18, 250 × 4 mm, 5 µm particle size), UV-VIS detector Sapphire, and Vacuum Degasser DG 3014 (ECOM, Prague, Czech Republic). The gradient was from 100% solvent A (acetonitrile/methanol/water—80/12/10, *v*/*v*/*v*), to 100% solvent B (methanol/ethylacetate, 95/5, *v*/*v*), both solvent mixtures contained 0.01% of BHT. The total analysis time was 25 min., the gradient run during 2–5 min, the flow rate was 1 mL·min^−1^; detection at 445 nm. Quantification of detected carotenoids was performed with Clarity software (DataApex, Prague, Czech Republic). The carotenoid content was expressed in micrograms of the particular carotenoid per gram of dry leaves (µg·g^−1^ DW). Standards of carotenoids were isolated from tobacco leaves, and individual carotenoids were separated and purified by HPLC with the same device as described above. Carotenoids purity measured on HPLC was 95–98%. Carotenoid standards were quantified by spectrophotometer Hitachi 2200 (Hitachi Ltd., Tokyo, Japan) using absorption coefficients.

### 2.6. Fatty Acid Methyl Esters (FAME) Extraction

For the determination of fatty acid content, the method published by DeLong et al. [15] was slightly modified. Freeze-dried homogenized tissue (10 mg) was extracted in 2 mL of 1N methanolic HCl and incubated at 80 °C for 60 min. Next, the samples were cooled down to room temperature, and 2 mL of 5% (*w*/*v*) sodium chloride and 1 mL of hexane was added, shaken, and centrifuged at 430× *g*. The supernatant was collected and the pellet re-extracted again in 1 mL of hexane. The procedure was repeated and combined supernatants were filtered through 2 mm column of anhydrous sodium sulfate to remove residual water. The column was washed with hexane and eluents were concentrated under nitrogen flow at 30 °C to 25 µL. Samples were re-dissolved in 500 µL of hexane and stored in −20 °C until analysis.

### 2.7. GC × GC-TOFMS Analysis of FAME

GC × GC-TOFMS analysis was performed on LECO Pegasus 4D system (Leco Corporation, St. Joseph, MI, USA) containing Agilent 7890 gas chromatograph (Agilent Technologies, Santa Clara, CF, USA) with a LECO dual-jet thermal modulator; Gerstel MultiPurpose Sampler and temperature-programmed CIS4 inlet (Gerstel GmbH & Co. KG, Mülheim an der Ruhr, Germany).

Gas chromatograph was fitted with precolumn—SGE Analytical Science BPX-5, non-polar, 5% phenyl polysilphenylene-siloxane (2.752 m × 0.25 mm I.D. × 0.25 µm film); primary column—SGE Analytical Science BPX5, non-polar, 5% phenyl polysilphenylene-siloxane (28.863 m × 0.25 mm I.D. × 0.25 µm film), modulatory column—SGE Analytical Science BPX5 (0.1 m × 0.25 mm I.D. × 0.25 µm film), secondary column—SGE Analytical Science BPX50, moderately-polar, 50% phenyl polysilphenylene-siloxane (0.833 m × 0.25 mm I.D. × 0.25 µm film) and transfer line—SGE Analytical Science BPX50 (0.21 m × 0.25 mm I.D. × 0.25 µm film).

Prior to injection, samples were diluted 10 times and internal standard methyl heptadecanoate-d_33_ was added (final concentration 1 ppm). The injection was performed under the following conditions: injection volume 1 µL + 1 µL air below, baffled liner, CIS4 inlet at −20 °C, initial time 0.05 min, ramp 10 °C/s to 250 °C, hold time 10 min.

The analysis was performed under the following conditions: splitless mode, helium BIP (purity 5.7, Air Products, Decin, Czech Republic) was used as a carrier gas at 1.4 mL·min^−1^, primary oven temperature 30 °C (5 min hold), ramp 10 °C/min until 285 °C (5 min hold), modulation period 1.8 s, 0.4 s hot pulse, 0.5 s cold pulse, modulator temperature program 15 °C above secondary column program, chiller at −80 °C, secondary column operated 5 °C above primary column program, transfer line at 280 °C.

Time of flight mass detector (EI) was operated under the following conditions: ion source at 250 °C, solvent delay 480 s, spectra collected between 14–600 amu at 100 Hz, detector voltage 1700 V, and electron energy −70 V. Whole analysis took 35.5 min.

Leco ChromaTOF^®^ software was used for data analysis and quantification. The 15-point calibration curve was measured for 36 individual FAMEs using Supelco 37 component FAME Mix (Sigma-Aldrich s.r.o., Prague, Czech Republic) as the external standard. Good linearity was achieved within ranges 0.0015 ppm to 29.97 ppm for individual FAMEs (coefficients of determination R^2^ between 0.990 and 0.9997). Obtained results were corrected using the internal standard concentration in each sample. Fatty acid (FA) concentration was calculated using conversion factor (cf) FAME–FA (cf = MW_FA_/MW_FAME_), and the content was expressed in micrograms per gram of dry leaves (µg·g^−1^ DW).

### 2.8. Statistical Evaluation

All analyses were performed at least from three biological samples, and three to five independent measurements were performed according to a particular assay. Results were expressed as means ± SD (standard deviation). Data were analyzed by STATISTICA software (StatSoft, TIBCO Software Inc., Prague, Czech Republic), subjected to one-way analysis of variance (ANOVA). Duncan’s test with a significant difference at *p* ≤ 0.05 level was used to separate the means. The Pearson correlation coefficient was calculated in order to evaluate the relationship between antioxidant capacity measured by ORAC assay and the content of antioxidant compounds (the total phenol content, or the carotenoid content, or the ascorbate content). The value of the Pearson correlation coefficient—*r* was expressed as follows: *r*(degress of freedom) statistics; *p* value. The results were significant at *p* < 0.05.

## 3. Results and Discussion

The search for European fern species was carried out in garden centers and botanical gardens. In addition, two species, *Pteridium aquilinum* and *Lastrea limbosperma*, were collected in the wild. *P. aquilinum* belongs to the most abundant edible ferns with cosmopolitan distribution [3]. Wild type of *L. limbosperma* was collected in order to detect possible variations caused by cultivation conditions (soil vs. wild). All in all, 24 fern species belonging to 8 families were included in this study. To compare obtained results with commonly consumed green vegetables, rocket (*Eruca sativa*) and spinach (*Spinacia oleracea*) were chosen as references.

### 3.1. Antioxidants in Fern Fiddleheads

For the evaluation of total phenol content and antioxidant capacity, methanolic extracts of dried fern fiddleheads were utilized. Colorimetric assay was employed for the assessment of total phenol content (Table 1). The average total phenol content was 232.6 mg GA_eqv_·g^−1^ DW and the highest content was determined in *Dryopteris dilatata*, 434.3 mg GA_eqv_·g^−1^ DW. When compared to the reference vegetables, fern fiddleheads contained on average 5 times higher amount of phenolic compounds, and almost 10 times in case of *D. dilatata*. Even the fern with the lowest total phenol content (*^H^Osmunda regalis*, 66.6 mg GA_eqv._·g^−1^ DW) had slightly higher phenol content than the reference vegetables (43.7 mg GA_eqv._·g^−1^ DW in average). The total phenol content positively correlated with the determined antioxidant capacity using ORAC assay (Table 1) with the value of Pearson correlation coefficient *r*(28) = 0.7881; *p* = 0.00001. Such correlation was also reported in other studies [16,17,18,19,20]. More than half of the examined fern species displayed remarkable antioxidant capacity exceeding the antioxidant capacity of the reference compound, Trolox (>1.0 g T_eqv_·g^−1^ DW, which equals to 4mM T_eqv_·g^−1^ DW). The lowest antioxidant capacity from the fern species was detected for *A. scolopendrium* (0.267 g T_eqv_·g^−1^ DW), which was still almost two times higher than the antioxidant capacity of the reference vegetables (0.15 g T_eqv_·g^−1^ DW in average). Furthermore, the average antioxidant capacity for all fern fiddleheads was 1.02 g T_eqv_·g^−1^ DW, with the highest value detected for *O. sensibilis* (1.95 g T_eqv_·g^−1^ DW). Interestingly, young fern fiddleheads analyzed in this study reached significantly higher antioxidant capacity in almost all tested species when compared to mature fern leaves that were subjected to analyses in our previous study [21]. In mature fern leaves, the average total phenol content was 163 mg GA_eqv_·g^−1^ DW and the average antioxidant capacity determined by ORAC assay was 0.675 g T_eqv_·g^−1^ DW.

Another valuable antioxidant compound present in plants is ascorbic acid (vitamin C), which is an essential vitamin for humans and needs to be acquired through diet [22]. It serves as a cofactor of many enzymes, thus maintaining their normal function, which ensures biosynthesis of carnitine and adrenal and functioning of fibroblasts and osteoblasts [22]. Therefore, the content of ascorbate in fern fiddleheads was measured. In general, the content of ascorbate in fiddleheads (637.4 μg·g^−1^ FW in average) was comparable to its content in the reference vegetables (542 µg·g^−1^ FW in average) as well to the content of other, commonly eaten, fruits and vegetables [22]. There were few species with significantly higher amount of ascorbate than in the reference vegetables, such as *A. filix-femina* (1565 μg·g^−1^ FW), *D. remota* (1329 μg·g^−1^ FW), or *D. dilatata* (1181 μg·g^−1^ FW), *D. carthusiana* (1157 μg·g^−1^ FW), and *D. aemula* (1115 μg·g^−1^ FW). From these results, it was obvious that *Athyrium* and *Dryopteris species* were distinguished by high ascorbate content (1253 μg·g^−1^ FW and 781 μg·g^−1^ FW in average, respectively). However, there was a very weak insignificant relationship between the content of ascorbate and the antioxidant capacity determined by ORAC assay, with the value of Pearson correlation coefficient *r*(25) = 0.009; *p* = 0.966.

### 3.2. Carotenoids and Carotenoid Profile in Fern Fiddleheads

Carotenoids also belong to antioxidants, and their high consumption, especially of lutein and zeaxanthin, has been reported to be beneficial to retinal tissue, as high concentrations of these pigments are present in the macular region of the human eye [23,24,25,26,27]. Thus, the presence of individual carotenoids in fiddlehead extracts was determined. The total content of carotenoids (Table 2) in fiddleheads (349 μg·g^−1^ DW in average) was comparable to the carotenoid content in the reference vegetable, *Eruca sativa* (385 μg·g^−1^ DW). The content of carotenoids in the other reference vegetable, *Spinacia oleracea*, was much lower (122 μg·g^−1^ DW). Thus, the total content of carotenoids in fern fiddleheads is not extraordinarily high and would be comparable to some green leafy vegetables. Moderate content of carotenoids also indicates that carotenoids do not contribute significantly to the antioxidant capacity of fern fiddleheads and the value of the Pearson correlation coefficient also suggested a weak insignificant relationship (*r*(28) = 0.0799; *p* = 0.686). In our previous study, the content of individual carotenoids in mature fern leaves was determined [21] and it was generally about 1.4 times higher than the content determined here in fiddleheads. In fiddleheads, the highest content of all carotenoids, above 500 μg·g^−1^ DW, was observed for two fern species, horticultural species of *Polystichum aculeatum* (560 μg·g^−1^ DW), and *Polystichum setiferum* (537 μg·g^−1^ DW). In horticultural species of *Polystichum aculeatum* were also detected the highest amounts of neoxanthin (71.0 μg·g^−1^ DW) and antheraxanthin (16.6 μg·g^−1^ DW; both supplementary Table S2), and high amounts of lutein (231.7 μg·g^−1^ DW) and *β*-carotene (186.2 μg·g^−1^ DW), while *Polystichum setiferum* had the highest content of lutein (252.4 μg·g^−1^ DW) and second highest content of violaxanthin (84.2 μg·g^−1^ DW). On average, fern extracts contained an insignificantly higher amount of *β*-carotene (112.7 μg·g^−1^ DW) than *Eruca sativa* (96.7 μg·g^−1^ DW). The highest content of *β*-carotene was present in *Dryopteris oreades* (201.6 μg·g^−1^ DW), and in horticultural species of *Polystichum aculeatum* (186.2 μg·g^−1^ DW). *β*-Carotene is a source of vitamin A, which protects the skin from excessive light radiation [28]. In addition, *β*-carotene is indispensable for the biosynthesis of the eye pigment, rhodopsin, as well as for the activation of rhodopsin, thus serving as a sensitizing chromophore for UV light [29]. In addition, the intake of naturally occurring carotenoids usually has a greater protective effect than the intake of synthetic supplements with *β*-carotene, which often blocks the absorption of natural carotenoids [30].

### 3.3. Antioxidants in Human Diet and Possible Contribution of Ferns

It is believed that the consumption of foods with high antioxidant capacity, such as fruits and herbs, may reduce the risk of age-related diseases associated with high levels of oxidative stress [31,32]. When the antioxidant capacity of ferns is compared to commonly eaten foods with high antioxidant content, it shows fern superiority. For example, Ninfali et al. [33] determined total phenol content and antioxidant capacity of various herbs, spices, and vegetables and the highest antioxidant capacity exerted cumin, 768 μM T_eqv_·g^−1^ DW (192 mg T_eqv_·g^−1^ DW). In another study performed by Kratchanova et al. [18], peppermint and thyme exhibited the highest values of antioxidant capacity from 25 medicinal plants studied, with 729 and 408 mg T_eqv_·g^−1^ DW, respectively. Moreover, the antioxidant capacity of methanolic extracts of 55 medicinal plants was evaluated by Wojcilkowski et al. [34] with the highest value obtained for *Olea europaea* (olives), 215 mg T_eqv_·g^−1^ DW. Even fruits and berries, which are considered rich sources of antioxidant compounds, exert much lower antioxidant capacity when compared to ferns. In the study of Wolfe et al. [35], the extracts of blueberries, strawberries, and cranberries showed the highest antioxidant capacity of 241, 210, and 209 mg T_eqv_·g^−1^ DW (recalculated from fresh weight (FW) assuming dry weight to be about 10% FW). On the other hand, the average antioxidant capacity determined here in fern fiddleheads was 1020 mg T_eqv_·g^−1^ DW.

The total phenol content and the antioxidant capacity of fern species was also evaluated by other authors [11,17,19]. In general, our results revealed slightly higher total phenol content and antioxidant capacity. For example, methanolic extracts of *Athyrium filix-femina*, *Dryopteris affinis*, and *D. filix-mas* were reported to contain between 88.7 and 234 mg GA_eqv_·g^−1^ DW of phenols, and their antioxidant capacity was between 320 and 1055 mg T_eqv_·g^−1^ DW (values were again recalculated from FW with the projection of 10% DW in FW). In addition, other methanolic extracts of *Asplenium trichomanes*, *A. caterach*, *Ceterach officinarum,* and *Polypodium vulgare* were reported to contain between 100 and 193 mg GA_eqv_·g^−1^ DW of phenols, with the antioxidant capacity being between 562 and 732 mg T_eqv_·g^−1^ DW [17]. On the other hand, our values were between 67 and 434 mg GA_eqv_·g^−1^ DW for phenols and between 267 and 1949 mg T_eqv_·g^−1^ DW for the antioxidant capacity. Similarly lower values than those obtained in our study were reported for the total phenol content and the antioxidant capacity of *Matteuccia struthiopteris* (51.6 vs. 248 mg GA_eqv_·g^−1^ DW for phenols and 382 vs. 1109 mg T_eqv_·g^−1^ DW for the antioxidant capacity) [11]. The observed differences may be caused by the different time of fiddleheads’ collection.

In *M. struthiopteris* also the ascorbate content along with the carotenoid content was evaluated. On the contrary to the phenol content, the reported values were higher than those detected in our study. In case of the ascorbate level, our value was about half the value reported (1.63 μmol·g^−1^ FW vs. 3.0 μmol·g^−1^ FW) [11]. On the other hand, similar content of ascorbate (1.68 μmol·g^−1^ FW) was detected in green vegetable *Asparagus officialis* [36]. In the case of the carotenoid content, our results gave lower amounts of all carotenoids and especially of two xanthophylls, violaxanthin, and zeaxanthin, when compared to the study of DeLong et al. [11]. They reported the amounts to be 225 and 127.4 μg·g^−1^ DW for violaxanthin and zeaxanthin, respectively, while our values were only 56.9 and 2.54 μg·g^−1^ DW, respectively. Similarly, very high amounts of lutein and neoxanthin were reported to be present in *Adiantum capillus*-*veneris* (806 and 143 μg·g^−1^ DW, respectively), while our values were in average 157 and 37 μg·g^−1^ DW [37]. On the other hand, except for violaxanthin and *β*-carotene-5,6-epoxide, they did not report any other carotenoids to be present. On the contrary, when the average total carotenoid content in ferns was compared to that of asparagus, it was about two times higher [36]. Similarly, water fern *Azolla filiculoides* had on average, about three times lower content of carotenoids than the fern species tested by us [38]. However, the carotenoid content depends immensely on environmental conditions (altitude, humidity, temperature) as well as the potential stress conditions (drought), which may account for this difference.

### 3.4. Fatty Acid Content and Profile in Fern Fiddleheads

Ferns are also known to be a valuable source of essential fatty acids. First studies on fatty acid composition in ferns were reported as early as 1975 [10,39]. In this study, the quantification of the total and individual content of fatty acids in fern fiddleheads was carried out. The content of individual fatty acids was determined as an average in fiddleheads of all fern species (Table 3). The total content of fatty acids (Table 4) was determined for each fern species together with the content of monounsaturated fatty acids (MUFA), polyunsaturated fatty acids (PUFA), saturated fatty acids, and omega-6/omega-3 fatty acid ratio (n6/n3). In fern fiddlehead extracts, about 30 different fatty acids were detected. The fatty acids present in the highest amount were linoleic acid (18:2, omega-6, 4595 mg·kg^−1^ DW), arachidonic acid (20:4, omega-6, 3844 mg·kg^−1^ DW), oleic acid (18:1, omega-9, 3121 mg·kg^−1^ DW), *α*-linolenic acid (18:3, omega-3, 2920 mg·kg^−1^ DW), palmitic acid (16:0, saturated, 1670 mg·kg^−1^ DW), and *γ*-linolenic acid (18:3, omega-6, 1617 mg·kg^−1^ DW). Such composition is similar to the composition reported by Jamieson and Reid [10], where *α*-linolenic acid was detected in the highest amount, followed by palmitic acid, linoleic acid, arachidonic acid, and oleic acid. In their work, also a high amount of hexadecatrienoic omega-3 fatty acid was measured, which was not detected in our fern samples. Likewise, Nekrasov et al. [40] found the following acids as the major ones in 23 fern species: *α*-linolenic, oleic, linoleic, palmitic, and arachidonic. They also reported that the fatty acid composition varied a lot among species as well as within the species as it is highly dependent on the presence of sporangia. The presence of sporangia increased the amount of linoleic and oleic acids and decreased the amount of *α*-linolenic and arachidonic acids. This is in agreement with reports that fern spores contain predominantly oleic and linoleic acids [41,42]. Spores are known to contain high levels of lipids, thus, their presence not only affected the fatty acid composition, but also the total content of lipids, which increased [43]. In addition, Nekrasov et al. [40] studied the differences in fatty acid content depending on the vegetation period. In the spring, young fronds (fiddleheads) contained linoleic and oleic acids in the highest amount, while in mature fronds, collected in summer, *α*-linolenic acid dominated. They accounted for these changes due to the accumulation of chloroplast membranes in green tissue. The absolute content of arachidonic acid was highest in young fronds without sporangia.

Interestingly, the reference vegetables, rocket, and spinach, contained only trace amounts of arachidonic acid, and zero amount of *γ*-linolenic and dihomo-*γ*-linolenic acids (DGLA). All three FAMEs belonged to omega-6 fatty acids and were detected in ferns in significant amounts. The content of particular FAMEs in individual samples is presented in supplementary Tables S3–S7.

Fatty acids (lipids) are important for humans as they mediate signaling pathways and form cell membranes. Omega-3 (n-3) and omega-6 (n-6) polyunsaturated fatty acids belong to essential fatty acids that human bodies cannot synthesize. From these, especially *α*-linolenic (n-3) and linoleic (n-6) acids are obtained through diet when eating fish and fish oils. However, with the depletion and contamination of marine fisheries, the difficulties with the cultivation of human algae, and the increasing number of vegan and vegetarian people, new sources of these fundamental fatty acids are sought [40]. Dietary intake of these essential fatty acids protects against mental and cardiovascular disorders, cancer, osteoporosis or diabetes [44]. The recommended consumption of fat in the human diet is 25%, however, the relative content of various fatty acids is also an important factor. For PUFAs, the recommended n-6/n-3 ratio is between 4:1 or lower in order to ensure the synthesis of enough eicosapentanoid acid. At a higher ratio, the syntheses of arachidonic acid are otherwise preferred [11]. The proportion of PUFAs in consumed fat should be around 11% [44]. Most of the fern species studied contained PUFAs with the desired n-6/n-3 ratio. Even the lowest n6/n3 ratio (around 2) was still about five times higher than the ratio in the reference vegetables (0.36 on average). From these results, it may be concluded that most of the commonly eaten green vegetables would lack a sufficient amount of omega-6 fatty acids. In addition, when the total content of fatty acids was compared on average, it was higher in fern species than in the reference vegetables by 1.6-fold, making fern fiddleheads a rich source of fatty acids. Moreover, the analysis of the fatty acid composition of common vegetable oils revealed that they are destitute of n-3 fatty acids no matter how rich in PUFAs they are. Thus, the high consumption of plant oils with low consumption of fish products, commonly seen in Western counties, provides the human diet with an unfavorable n-6/n-3 ratio [44].

### 3.5. Fern Toxicity

When considering ferns as prospective widely available vegetables, a caution should be paid to their potential toxicity. Some fern species are known to contain a range of toxic compounds. In this respect, the most studied and discussed is the fern species *Pteridium aquilinum* (bracken). Bracken is one of the five most common plants on the planet, and in many countries, such as Japan, Brasil, Canada, and China, its fiddleheads have long been consumed as a delicious food [45,46]. However, these young unfolded fronds were found to exhibit mutagenic, teratogenic, clastogenic, and carcinogenic activities, which have been attributed to the presence of a nor-sesquiterpene illudane glycoside, ptaquiloside. In addition, 24 different sesquiterpenes, many of illudane structure with a reactive cyclopropane ring, were isolated from bracken, which may also contribute to bracken toxicity [47]. These toxic effects were mainly observed in domestic animals grazing on lands largely infested with bracken. In humans, these effects are not as pronounced, probably due to the way bracken is treated before eating [46]. Nevertheless, in countries with a bracken-rich diet, a high incidence of gastric, esophageal, or pancreatic cancer has been observed. On the other hand, ptaquiloside was found to be highly soluble in water and have low-temperature stability and mutable stability at various pH [47]. Thus, if known procedures of bracken processing are followed and it is not consumed excessively, the potential risk of diverse effects may be minimalized. Conversely, a greater risk may arise from the contamination of milk by ptaquiloside and related compounds in areas where cattle graze on poor pastures infested with bracken, despite the finding that the amount of ptaquiloside in milk may be lowered by its pasteurization [45,46]. In addition, the contamination of ground, surface, and well waters near bracken sites was detected [47].

In the work of Saito et al., ptaquiloside or its analogues were detected in 19 out of 31 ferns, especially in those of genera *Pteris*, *Microlepia*, and *Hypolepis* [48]. On the other hand, among the 21 species from Denmark, ptaquiloside was only found in *Pteridium aquilinum* [49]. Many of the species studied in Denmark were also included in our study. In addition, the toxicity of mature leaf extracts of all the species reported here was previously tested by us on sheep hepatocytes at 100 µg·mL^−1^ and they were found to be non-toxic [21].

When the toxicity of other fern species is taken into account, it seems that those ptaquiloside-free are either non-toxic or exhibit low toxicity, though the number of such studies is rather limited. For example, the toxicity of an aqueous extract of *Polypodium leucomotos*, which is marketed as Fernblock^®^ for oral and topical photoprotection, was evaluated. No toxicity (genotoxicity, mutagenicity, oral toxicity) was observed in this case [50,51]. In the case of *Macrothelypteris torresiana*, no acute toxicity was observed at 2000 mg·kg^−1^, along with no changes in hematological and biochemical parameters [52]. The acute and sub-chronic toxicity of *Dryopteris filix-mas* ethanol leaf extract was evaluated [53]. From the genus *Dryopteris*, whose extracts and rhizomes were used in the past as anthelmintic agents, unusual acylphloroglucinols (albaspidins, pentherin-I, flavaspidic, and filixic acids) were isolated [54,55]. Such acylphloroglucinols were found to cause drowsiness and blindness in cattle eating rhizomes of *D. filix-max* [55]. However, the acute toxicity test of *D. filix-mas* extract using albino rats did not show toxicity or death at 5000 mg·kg^−1^. And although the sub-chronic evaluation revealed potential hepatotoxicity and nephrotoxicity of the extract when used for long periods at 250 and 500 mg·kg^−1^, these toxicities were reversible in recovery studies [53]. Similarly, no acute toxicity was observed for *Dryopteris crassirhizoma* below 2000 mg·kg^−1^, along with no genotoxicity observed in bacterial reverse mutation, chromosomal aberration, and bone marrow micronucleus tests [56].

All in all, if fern fiddleheads are not eaten on a daily basis and are consumed dried or appropriately cooked, the risk of potential intoxication is reasonably lowered. In addition, most of the species grown in Europe seem to be devoid of toxicity and toxic compounds (ptaquiloside and its analogues), thus rendering them as prospective vegetables.

## 4. Conclusions

In summary, fiddleheads from European ferns may contribute to the human diet as a rich source of valuable antioxidants and essential fatty acids with a desirable n-6/n-3 ratio. Our work may serve as a guideline for the selection of suitable fern species to be grown commercially as vegetables. *Polystichum aculeatum*, *Polypodium vulgare*, or *Onoclea sensibilis* are examples of species with high total phenol content and antioxidant capacity together with a preferential n-6/n-3 ratio. Although the concern regarding the possible toxicity still needs to be clarified and evaluated via more thorough assays, a considerate consumption of fern fiddleheads together with their proper cooking or processing before eating should avoid potential poisoning. For ferns, as for other foods (even those so-called “superfoods”), “moderation in all things” applies. We believe that fern fiddleheads may variegate the vegetable range available in Europe, plus, consumers would gain access to local nutrient-dense food.

## Figures and Tables

**Table 1:** The content of antioxidants and antioxidant capacity in fern fiddleheads.

		Total Phenol Content				ORAC				Ascorbate			
Species	Family	(mg GA_eqv_·g^−1^ DW)				TE (mg·g^−1^ DW)				(µg.g^−1^ FW)			
*^H^* *Asplenium scolopendrium*	*Aspleniaceae*	79.9	±	4.5		267	±	41.9		ND			
*Athyrium distentifolium*	*Athyriaceae*	398.7	±	45.6	˃1.2M	1315	±	266.9	˃1.2M	942	±	131.9	*
*^H^* *Athyrium filix-femina*	*Athyriaceae*	234.5	±	30.2		822	±	167.5		1565	±	33.5	*
*^W^* *Pteridium aquilinum*	*Dennstaedtiaceae*	81.9	±	13.1		476	±	88.7		597	±	40.5	
*Dryopteris aemula*	*Dryopteridaceae*	233.2	±	17.2		500	±	85.1		1115	±	112.2	*
*Dryopteris affinis*	*Dryopteridaceae*	97.9	±	9.9		644	±	137.3		319	±	40.5	
*Dryopteris borreri*	*Dryopteridaceae*	323.2	±	32.1	˃1.2M	1095	±	178.2		557	±	5.4	
*Dryopteris cambrensis*	*Dryopteridaceae*	343.3	±	40.3	˃1.2M	1116	±	241.0		821	±	24.0	*
*Dryopteris carthusiana*	*Dryopteridaceae*	272.4	±	33.3		874	±	141.8		1157	±	32.7	*
*Dryopteris caucasica*	*Dryopteridaceae*	232.1	±	36.2		1111	±	144.9		557	±	66.7	
*^H^* *Dryopteris dilatata*	*Dryopteridaceae*	434.3	±	66.6	* ˃1.2M	1355	±	275.4	˃1.2M	1181	±	127.1	*
*Dryopteris expansa*	*Dryopteridaceae*	265.5	±	30.9		1133	±	159.3		413	±	30.3	
*^H^* *Dryopteris filix-mas*	*Dryopteridaceae*	280.2	±	43.8	˃1.2M	1221	±	233.2	˃1.2M	690	±	11.6	
*Dryopteris oreades*	*Dryopteridaceae*	121.2	±	16.5		638	±	178.2		456	±	21.7	
*Dryopteris remota*	*Dryopteridaceae*	319.1	±	47.1	˃1.2M	1446	±	312.1	˃1.2M	1329	±	134.6	*
*^H^* *Polystichum aculeatum*	*Dryopteridaceae*	228.9	±	23.8		958	±	189.5		377	±	10.5	
*Polystichum aculeatum*	*Dryopteridaceae*	382.0	±	45.5	˃1.2M	1318	±	251.3	˃1.2M	322	±	6.2	
*Polystichum setiferum*	*Dryopteridaceae*	272.1	±	37.2		1317	±	241.3	˃1.2M	301	±	34.0	
*^H^* *Matteuccia struthiopteris*	*Onocleaceae*	247.9	±	39.5		1109	±	178.5		323	±	6.5	
*Onoclea sensibilis*	*Onocleaceae*	365.1	±	23.8	˃1.2M	1949	±	409.3	* ˃1.2M	ND			
*^H^* *Osmunda regalis*	*Osmundaceae*	66.6	±	7.3		328	±	55.2		511	±	13.0	
*Osmunda regalis*	*Osmundaceae*	123.7	±	15.9		719	±	138.2		494	±	4.1	
*^H^* *Polypodium vulgare*	*Polypodiaceae*	212.4	±	26.8		1410	±	230.5	˃1.2M	366	±	17.1	
*Polypodium vulgare*	*Polypodiaceae*	182.7	±	27.6		1573	±	285.6	˃1.2M	342	±	2.6	
*Lastrea limbosperma*	*Thelypteridaceae*	195.8	±	10.8		824	±	177.8		ND			
*^W^* *Lastrea limbosperma*	*Thelypteridaceae*	145.4	±	5.7		823	±	163.4		515	±	19.1	
*Phegopteris connectilis*	*Thelypteridaceae*	126.8	±	7.4		1009	±	142.9		353	±	15.5	
*Thelypteris palustris*	*Thelypteridaceae*	245.8	±	14.7		1210	±	270.3	˃1.2M	332	±	3.4	
Average of all ferns		*232.6*	±	26.9		*1020*	±	192.3		*637*	±	37.8	
*Eruca sativa*	*Brassicaceae*	58.3	±	1.0		175	±	41.5		442	±	29.5	
*Spinacia oleracea*	*Amaranthaceae*	29.0	±	3.7		125	±	38.7		642	±	41.3	

*^H^* marks horticulture species from garden centres, cultivated in pots, *^W^* marks species collected in wild, unmarked fern species were collected in botanical gardens, ND (not determined due to insufficient amount of plant material), * marks species statistically distinguished from all samples (ANOVA, Duncan test at *p* ≤ 0.05), ˃1.2 M marks values exceeding 1.2 times higher to median value.

**Table 2:** Content of total carotenoids and the major carotenoids lutein and *β*-carotene in fern fiddleheads analyzed by HPLC.

	Lutein				*β*-Carotene				Total Carotenoids				Ratio of *β*-Carotene to Total Carotenoids			
Species	(µg·g^−1^ DW)				(µg·g^−1^ DW)				(µg·g^−1^ DW)				Car/(X+C)			
*^H^* *Asplenium scolopendrium*	211.75	±	7.62		88.85	±	6.37		389.26	±	17.86		0.228	±	0.007	
*Athyrium distentifolium*	179.82	±	19.58		96.45	±	6.30		378.92	±	28.51		0.255	±	0.015	
*^H^* *Athyrium filix-femina*	223.24	±	28.27		154.61	±	11.13		478.16	±	20.24		0.324	±	0.034	
*^W^* *Pteridium aquilinum*	143.03	±	15.31		67.82	±	0.92		301.30	±	16.27		0.225	±	0.009	
*Dryopteris aemula*	117.53	±	14.65		98.80	±	7.34		262.82	±	23.57		0.376	±	0.010	
*Dryopteris affinis*	92.00	±	4.45		54.54	±	2.58		194.40	±	6.50		0.281	±	0.023	
*Dryopteris borreri*	59.81	±	4.36		100.03	±	5.91		214.32	±	13.54		0.467	±	0.011	˃1.5M
*Dryopteris cambrensis*	25.25	±	3.08		11.99	±	1.98		63.27	±	6.23		0.193	±	0.051	
*Dryopteris carthusiana*	241.03	±	45.23	˃1.5M	126.32	±	13.60		495.95	±	65.65		0.256	±	0.016	
*Dryopteris caucasica*	121.57	±	11.85		156.42	±	11.33	˃1.5M	360.21	±	23.93		0.434	±	0.013	˃1.5M
*^H^* *Dryopteris dilatata*	108.36	±	5.35		100.69	±	9.64		235.94	±	8.51		0.427	±	0.035	˃1.5M
*Dryopteris expansa*	158.37	±	41.43		180.10	±	19.15	* ˃1.5M	395.30	±	61.90		0.458	±	0.025	˃1.5M
*^H^* *Dryopteris filix-mas*	216.40	±	23.84		127.89	±	11.29		416.08	±	40.11		0.308	±	0.003	
*Dryopteris oreades*	98.63	±	9.57		201.61	±	7.03	* ˃1.5M	406.46	±	15.07		0.496	±	0.005	* ˃1.5M
*Dryopteris remota*	112.40	±	9.88		156.83	±	7.86	˃1.5M	312.04	±	20.87		0.503	±	0.011	* ˃1.5M
*^H^* *Polystichum aculeatum*	231.69	±	25.98		186.18	±	11.06	* ˃1.5M	560.11	±	11.29	˃1.5M	0.333	±	0.022	
*Polystichum aculeatum*	161.62	±	2.63		179.93	±	9.08	* ˃1.5M	447.42	±	15.97		0.402	±	0.007	
*Polystichum setiferum*	252.42	±	24.73	˃1.5M	127.17	±	13.22		536.59	±	45.18		0.237	±	0.005	
*^H^* *Matteuccia struthiopteris*	207.08	±	10.56		92.21	±	4.04		409.92	±	20.10		0.225	±	0.018	
*Onoclea sensibilis*	107.55	±	14.04		56.82	±	6.47		215.91	±	25.73		0.263	±	0.007	
*^H^* *Osmunda regalis*	182.53	±	22.79		109.16	±	8.43		364.13	±	9.21		0.300	±	0.030	
*Osmunda regalis*	162.62	±	6.29		91.24	±	2.79		321.83	±	11.92		0.284	±	0.006	
*^H^* *Polypodium vulgare*	229.13	±	15.17		137.18	±	8.19		464.09	±	18.17		0.295	±	0.009	
*Polypodium vulgare*	196.24	±	10.56		109.17	±	7.42		405.65	±	12.98		0.269	±	0.015	
*Lastrea limbosperma*	141.36	±	0.74		64.17	±	8.95		286.15	±	13.33		0.224	±	0.020	
*^W^* *Lastrea limbosperma*	223.13	±	15.83		101.29	±	6.43		449.63	±	24.76		0.225	±	0.002	
*Phegopteris connectilis*	93.09	±	8.19		53.45	±	2.29		198.42	±	15.02		0.270	±	0.009	
*Thelypteris palustris*	98.37	±	10.45		49.84	±	1.20		209.47	±	15.30		0.239	±	0.022	
Average of all ferns	*157.00*	±	14.73		*110.03*	±	7.57		*349.06*	±	21.70		*0.314*	±	0.016	
*Eruca sativa*	188.26	±	22.75		96.68	±	12.14		384.95	±	42.30		0.251	±	0.005	
*Spinacia oleracea*	68.86	±	10.61		30.58	±	3.50		122.09	±	17.50		0.251	±	0.009	

*^H^* marks horticulture species from garden centers, cultivated in pots, *^W^* marks species collected in wild, unmarked fern species were collected in botanical gardens, * marks species statistically distinguished from all samples (ANOVA, Duncan test at *p* ≤ 0.05), ˃1.5 M marks values exceeding 1.5 times higher to the median value.

**Table 3:** The average content of individual FAMEs in all tested fern fiddlehead samples.

No	Fatty Acid	µg.g^−1^ DW				Class
1	Caproic A (C6:0)	21.82	±	3.31		saturated
2	Caprylic A (C8:0)	2.75	±	1.16		saturated
3	Capric A (C10:0)	3.92	±	1.20		saturated
4	Lauric A (C12:0)	6.06	±	1.14		saturated
5	Myristoleic A (C14:1n5)	8.70	±	2.14		omega-5 MUFA
6	Myristic A (C14:0)	23.59	±	9.51		saturated
7	Pentadecanoic A (C15:0)	20.76	±	7.72		saturated
8	Palmitoleic A (C16:1n7)	454.14	±	240.59		omega-7 MUFA
9	Palmitic A (C16:0)	1670.22	±	327.07	* ˃10M	saturated
10	cis-10-Heptadecenoic A (C17:1n7)	16.21	±	3.53		omega-7 MUFA
11	Margaric A (C17:0)	43.52	±	7.61		saturated
12	gamma-Linolenic A (C18:3n6)	1617.27	±	438.11	* ˃10M	omega-6 PUFA
13	alfa-Linolenic A (C18:3n3)	2920.19	±	889.99	* ˃10M	omega-3 PUFA
14	Linoleic A (C18:2n6c)	4595.08	±	1122.70	* ˃10M	omega-6 PUFA
15	Oleic A (C18:1n9c)	3120.81	±	701.50	* ˃10M	omega-9 MUFA
16	Stearic A (C18:0)	127.32	±	53.79		saturated
17	Timnodonic A (C20:5n3) - EPA	580.99	±	433.38	˃10M	omega-3 PUFA
18	Arachidonic A (C20:4n6)	3844.45	±	939.00	* ˃10M	omega-6 PUFA
19	DGLA (C20:3n6)	878.55	±	326.11	˃10M	omega-6 PUFA
20	Eicosatrienoic A (C20:3n3) - ETE	17.94	±	8.10		omega-3 PUFA
21	Eicosadienoic A (C20:2n6)	48.54	±	17.89		omega-6 PUFA
22	Gondoic A (C20:1n9)	177.54	±	110.29		omega-9 MUFA
23	Arachidic A (C20:0)	108.87	±	68.74		saturated
24	Heneicosanoic A (C21:0)	6.17	±	3.61		saturated
25	Cervonic A (C22:6n3) - DHA	19.58	±	6.26		omega-3 PUFA
26	Docosadienoic A (C22:2n6)	6.75	±	3.31		omega-6 PUFA
27	Erucic A (C22:1n9)	14.58	±	11.69		omega-9 MUFA
28	Behenic A (C22:0)	267.97	±	126.75		saturated
29	Tricosanoic A (C23:0)	20.19	±	12.09		saturated
30	Nervonic A (C24:1n9)	100.62	±	80.68		omega-9 MUFA
31	Lignoceric A (C24:0)	152.08	±	38.69		saturated

DGLA = dihomo-gamma-linolenic acid; MUFA = monounsaturated fatty acid; PUFA = polyunsaturated fatty acid, * marks FAMEs statistically distinguished in the content from all (ANOVA, Duncan test at *p* ≤ 0.005), ˃10 M marks values exceeding 10 times higher to the median value.

**Table 4:** Total content of FAMEs in fiddleheads of tested individual fern species.

	Total Content	PUFA/Satur.	n6/n3	MUFA	PUFA	Saturated
Species	µg.g^−1^ DW	Ratio	Ratio	µg.g^−1^ DW	µg.g^−1^ DW	µg.g^−1^ DW
*^H^* *Asplenium scolopendrium*	14,713	5.35	6.41	2790	10,044	1879
*Athyrium distentifolium*	22,094	6.74	2.45	4180	15,599	2316
*^H^* *Athyrium filix-femina*	22,527	6.90	3.41	3692	16,452	2383
*^W^* *Pteridium aquilinum*	24,032	5.67	4.80	3298	17,627	3107
*Dryopteris aemula*	21,501	5.82	3.27	4379	14,612	2510
*Dryopteris affinis*	13,563	5.29	3.56	2773	9074	1715
*Dryopteris borreri*	17,579	5.67	3.75	3566	11,911	2102
*Dryopteris cambrensis*	13,704	5.77	3.66	2969	9149	1587
*Dryopteris carthusiana*	18,661	5.59	3.20	3670	12,714	2276
*Dryopteris caucasica*	24,531	6.31	2.95	5412	16,505	2614
*^H^* *Dryopteris dilatata*	19,149	5.98	3.41	3919	13,048	2182
*Dryopteris expansa*	29,757	6.65	2.62	6932	19,842	2982
*^H^* *Dryopteris filix-mas*	19,446	6.14	3.05	3792	13,463	2191
*Dryopteris oreades*	18,984	6.74	3.48	3765	13,254	1965
*Dryopteris remota*	17,473	6.08	3.90	2932	12,487	2054
*^H^* *Polystichum aculeatum*	22,993	5.79	2.90	4299	15,940	2753
*Polystichum aculeatum*	17,693	5.00	4.46	3138	12,127	2427
*Polystichum setiferum*	20,487	5.15	3.40	4135	13,692	2660
*^H^* *Matteuccia struthiopteris*	24,216	6.77	2.33	4118	17,510	2588
*Onoclea sensibilis*	19,352	5.85	3.78	3271	13,734	2347
*^H^* *Osmunda regalis*	19,281	5.07	1.59	3153	13,472	2656
*Osmunda regalis*	17,545	4.75	1.27	2869	12,124	2551
*^H^* *Polypodium vulgare*	29,688	7.99	4.45	5198	21,765	2725
*Polypodium vulgare*	23,891	8.41	6.04	4292	17,516	2083
*Lastrea limbosperma*	19,621	5.12	2.91	3387	13,580	2654
*^W^* *Lastrea limbosperma*	23,965	5.34	1.98	4491	16,403	3071
*Phegopteris connectilis*	25,413	4.33	3.23	4311	17,144	3957
*Thelypteris palustris*	23,306	5.33	2.59	4264	16,034	3008
Average of all ferns	*20,899*	*5.91*	*3.39*	*3893*	*14,529*	*2477*
*Eruca sativa*	14,146	4.42	0.38	2174	9765	2208
*Spinacia oleracea*	10,834	6.06	0.34	1762	7787	1285

*^H^* marks horticulture species from garden centers, cultivated in pots; *^W^* marks species collected in wild; unmarked fern species were collected in botanical gardens; MUFA = monounsaturated fatty acid; PUFA = polyunsaturated fatty acid; n6 = omega-6 fatty acid; n3 = omega-3 fatty acid.

## Data Availability

Data is contained within this article.

## Supplementary Materials

The following are available online at https://www.mdpi.com/2304-8158/10/2/460/s1, Table S1: The list of fern species and reference vegetables, whose fiddleheads/young leaves were analysed, and source of plant material collection, Table S2: The content of minor xanthophylls in fern fiddleheads analysed by HPLC, Table S3: The average content of individual FAMEs in *Asplenium*, *Athyriaceae*, and *Dennstaedtiaceae* fern samples, Table S4, S5: The average content of individual FAMEs in *Dryopteridaceae* fern samples, Table S6: The average content of individual FAMEs in *Onocleaceae*, *Osmundaceae*, and *Polypodiaceae* fern samples, Table S7: The average content of individual FAMEs in *Thelypteridaceae* fern samples and samples of reference vegetables.
