# Supplementary material for: Nutritional and Antioxidant Potential of Fiddleheads from European Ferns
Source: Foods. 2021 Feb 19;10(2):460. doi: 10.3390/foods10020460 (PMC7923283; doi:10.3390/foods10020460)
Supplement: Supplementary file 1 [file foods-10-00460-s001.pdf]

Supplementary material

Table S1: The list of fern species and reference vegetables whose fiddleheads/young leaves were analysed, and source of plant material collection.

Species	Family	Source of plant material
[H]*Asplenium scolopendrium*	*Aspleniaceae*	Garden centre Krulich
Athyrium distentifolium	*Athyriaceae*	Fern collection garden in Telč
[H]*Athyrium filix-femina*	*Athyriaceae*	Garden centre Krulich
[W]*Pteridium aquilinum*	*Dennstaedtiaceae*	Wild collection (49°16´13.325 N; 15°22´46.346 E)
Dryopteris aemula	*Dryopteridaceae*	Fern collection garden in Telč
Dryopteris affinis	*Dryopteridaceae*	Fern collection garden in Telč
Dryopteris borreri	*Dryopteridaceae*	Fern collection garden in Telč
Dryopteris cambrensis	*Dryopteridaceae*	Fern collection garden in Telč
Dryopteris carthusiana	*Dryopteridaceae*	Botanical garden of the Charles University in Prague
Dryopteris caucasica	*Dryopteridaceae*	Fern collection garden in Telč
[H]*Dryopteris dilatata*	*Dryopteridaceae*	Garden centre Franc
Dryopteris expansa	*Dryopteridaceae*	Fern collection garden in Telč
[H]*Dryopteris filix-mas*	*Dryopteridaceae*	Garden centre Krulich
Dryopteris oreades	*Dryopteridaceae*	Fern collection garden in Telč
Dryopteris remota	*Dryopteridaceae*	Fern collection garden in Telč
[H]*Polystichum aculeatum*	*Dryopteridaceae*	Garden centre Franc
Polystichum aculeatum	*Dryopteridaceae*	Botanical garden of the Charles University in Prague
Polystichum setiferum	*Dryopteridaceae*	Fern collection garden in Telč
[H]*Matteuccia struthiopteris*	*Onocleaceae*	Garden centre Krulich
Onoclea sensibilis	*Onocleaceae*	Botanical garden of the Charles University in Prague
[H]*Osmunda regalis*	*Osmundaceae*	Garden centre Franc
Osmunda regalis	*Osmundaceae*	Botanical garden of the Charles University in Prague
[H]*Polypodium vulgare*	*Polypodiaceae*	Garden centre Franc
Polypodium vulgare	*Polypodiaceae*	Botanical garden of the Charles University in Prague
Lastrea limbosperma	*Thelypteridaceae*	Fern collection garden in Telč
[W]*Lastrea limbosperma*	*Thelypteridaceae*	Wild collection (49°13'14.214"N, 15°33'50.667"E)
Phegopteris connectilis	*Thelypteridaceae*	Botanical garden of the Charles University in Prague
Thelypteris palustris	*Thelypteridaceae*	Botanical garden of the Charles University in Prague
Eruca sativa	*Brassicaceae*	seedling, SEMO a.s. (Smržice, CZ)
Spinacia oleracea	*Amaranthaceae*	seedling, Seva Moravia s.r.o. (Valtice, CZ)

[H] = marks horticultural species from garden centres Krulich (Jakub Krulich, Prague, Czech Republic) and Franc (FRANC, Kamenné Žehrovice, Czech Republic) and were cultivated in pots; [W] = marks species collected in the wild, coordinates of the locality are stated in brackets; unmarked fern species were collected in private fern collection garden of RNDr. Libor Ekrt, Ph.D. in Telč (Czech Republic, coordinates: 49°11‘19.385‘‘N, 15°27‘11.701‘‘E), or in the Botanical Garden Charles University of Prague (Czech Republic, coordinates: 50°4’14.988‘‘N, 14°25’14.621‘‘E).

Table S2: The content of minor xanthophylls in fern fiddleheads analysed by HPLC.

Species	**Neoxanthin** (µg·g^{-1} DW)		**Violaxanthin** (µg·g^{-1} DW)		**Antheraxanthin** (µg·g^{-1} DW)		**Zeaxanthin** (µg·g^{-1} DW)	
^{H}*Asplenium scolopendrium*	47.01 ± 3.37		27.19 ± 1.20		10.18 ± 0.70		4.28 ± 0.41	>1.5M
Athyrium distentifolium	39.43 ± 4.37		48.60 ± 1.08		10.77 ± 1.15	>1.5M	3.85 ± 0.17	
^{H}*Athyrium filix-femina*	54.76 ± 4.18	>1.5M	34.03 ± 1.87		7.67 ± 0.16		3.84 ± 0.26	
^{W}*Pteridium aquilinum*	39.77 ± 3.05		40.85 ± 3.16		6.69 ± 0.37		3.14 ± 0.13	
Dryopteris aemula	23.04 ± 2.32		21.86 ± 3.15		1.16 ± 0.16		0.43 ± 0.05	
Dryopteris affinis	22.48 ± 3.06		20.62 ± 1.17		2.44 ± 0.30		2.32 ± 0.25	
Dryopteris borreri	23.11 ± 2.12		29.45 ± 2.92		0.72 ± 0.18		1.22 ± 0.21	
Dryopteris cambrensis	15.38 ± 4.44		8.73 ± 1.08		1.11 ± 0.24		0.79 ± 0.00	
Dryopteris carthusiana	53.52 ± 5.33	>1.5M	60.28 ± 7.15	>1.5M	10.70 ± 2.00	>1.5M	4.09 ± 0.51	>1.5M
Dryopteris caucasica	28.68 ± 2.14		42.32 ± 1.97		9.07 ± 0.56		2.15 ± 0.25	
^{H}*Dryopteris dilatata*	8.38 ± 2.32		13.73 ± 2.26		1.40 ± 0.12		3.38 ± 0.16	
Dryopteris expansa	27.41 ± 1.51		26.34 ± 3.18		1.43 ± 0.16		1.64 ± 0.13	
^{H}*Dryopteris filix-mas*	52.01 ± 4.87	>1.5M	14.87 ± 0.49		1.68 ± 0.26		3.23 ± 0.58	
Dryopteris oreades	52.11 ± 2.77	>1.5M	42.46 ± 1.71		8.99 ± 0.65		2.66 ± 0.49	
Dryopteris remota	17.13 ± 2.75		18.14 ± 0.82		5.46 ± 0.31		2.08 ± 0.29	
^{H}*Polystichum aculeatum*	70.95 ± 6.69	* >1.5M	51.45 ± 4.32	>1.5M	16.57 ± 1.07	* >1.5M	3.27 ± 0.37	
Polystichum aculeatum	53.99 ± 2.50	>1.5M	43.10 ± 3.22		6.78 ± 0.60		2.01 ± 0.66	
Polystichum setiferum	57.64 ± 5.31	>1.5M	84.19 ± 1.77	* >1.5M	12.15 ± 1.39	* >1.5M	3.02 ± 0.92	
^{H}*Matteuccia struthiopteris*	43.62 ± 5.02		56.91 ± 5.93	>1.5M	7.55 ± 0.88		2.54 ± 0.18	
Onoclea sensibilis	20.51 ± 2.59		19.28 ± 2.14		9.52 ± 0.96		2.23 ± 0.09	
^{H}*Osmunda regalis*	26.54 ± 4.22		32.06 ± 2.89		10.28 ± 0.96		3.56 ± 0.37	
Osmunda regalis	26.41 ± 1.63		27.75 ± 1.44		10.83 ± 0.51	>1.5M	2.99 ± 1.09	
^{H}*Polypodium vulgare*	52.12 ± 4.90	>1.5M	40.82 ± 2.84		3.47 ± 0.11		1.37 ± 0.32	
Polypodium vulgare	45.67 ± 2.17		47.46 ± 2.14		3.34 ± 0.32		3.77 ± 0.15	
Lastrea limbosperma	31.72 ± 1.97		39.15 ± 1.81		6.63 ± 0.37		3.12 ± 0.27	
^{W}*Lastrea limbosperma*	54.46 ± 4.05	>1.5M	53.65 ± 4.22	>1.5M	13.02 ± 0.68	* >1.5M	4.08 ± 0.23	>1.5M
Phegopteris connectilis	23.59 ± 2.68		21.69 ± 1.15		4.34 ± 0.73		2.26 ± 0.43	
Thelypteris palustris	25.31 ± 3.49		27.99 ± 5.40		5.75 ± 0.46		2.21 ± 0.23	
Average of all ferns	*37.03* ± 3.42		*35.53* ± 2.59		*6.78* ± 0.58		*2.70* ± 0.33	
Eruca sativa	41.73 ± 4.20		42.21 ± 2.57		12.35 ± 0.89	* >1.5M	3.72 ± 0.70	
Spinacia oleracea	14.67 ± 2.68		4.73 ± 1.71		2.34 ± 0.40		0.91 ± 0.11	

^{H} marks horticulture species from garden centres, cultivated in pots, ^{W} marks species collected in wild, unmarked fern species were collected in botanical gardens, * marks species statistically distinguished from all samples, >1.5M marks values exceeding 1.5 times higher to median value. Anova, Duncan test at $p \leq 0.05$

Table S3: The average content of individual FAMEs in *Aspleniaceae*, *Athyriaceae*, and *Dennstaedtiaceae* fern samples.

	Species/Family	[H]*Asplenium scolopendrium*			*Athyrium distentifolium*			[H]*Athyrium filix-femina*			[W]*Pteridium aquilinum*		
	FAME (µg.g^{-1} DW)	Aspleniaceae			Athyriaceae			Athyriaceae			Dennstaedtiaceae		
1	Caproic A (C6:0)	22.62	±	*4.63*	25.93	±	*0.33*	23.62	±	*2.59*	15.29	±	*7.60*
2	Caprylic A (C8:0)	3.29	±	*0.96*	3.31	±	*0.32*	3.19	±	*0.54*	3.41	±	*0.13*
3	Capric A (C10:0)	4.39	±	*1.03*	4.41	±	*0.53*	4.46	±	*0.72*	4.17	±	*0.68*
4	Lauric A (C12:0)	5.49	±	*0.75*	5.50	±	*0.49*	6.14	±	*0.25*	5.85	±	*0.77*
5	Myristoleic A (C14:1n5)	0.00	±	*0.00*	8.86	±	*1.34*	9.78	±	*0.02*	3.86	±	*5.46*
6	Myristic A (C14:0)	26.82	±	*1.78*	27.34	±	*0.48*	21.36	±	*5.80*	30.03	±	*1.30*
7	Pentadecanoic A (C15:0)	32.70	±	*2.25*	27.30	±	*0.23*	19.05	±	*1.61*	15.05	±	*0.05*
8	Palmitoleic A (C16:1n7)	302.82	±	*23.34*	490.41	±	*9.16*	475.76	±	*75.72*	234.01	±	*3.38*
9	Palmitic A (C16:0)	1266.89	±	*65.85*	1702.06	±	*14.44*	1743.98	±	*140.88*	2092.96	±	*58.48*
10	cis-10-Heptadecenoic A (C17:1n7)	13.73	±	*1.60*	18.14	±	*0.38*	14.71	±	*1.65*	9.84	±	*0.39*
11	Margaric A (C17:0)	32.67	±	*2.31*	39.94	±	*0.17*	41.25	±	*3.21*	30.46	±	*0.02*
12	gamma-Linolenic A (C18:3n6)	1191.81	±	*148.93*	2147.43	±	*40.00*	2122.30	±	*157.01*	2013.23	±	*41.23*
13	Linolenic A (C18:3n3)	1217.92	±	*68.05*	2886.11	±	*86.90*	3148.07	±	*214.28*	2512.28	±	*43.15*
14	Linoleic A (C18:2n6c)	3833.94	±	*252.56*	4733.47	±	*171.93*	4929.11	±	*374.99*	5904.59	±	*51.63*
15	Oleic A (C18:1n9c)	2118.44	±	*208.79*	3345.88	±	*78.30*	3042.41	±	*370.75*	2937.21	±	*206.44*
16	Stearic A (C18:0)	54.94	±	*6.55*	102.83	±	*2.67*	113.46	±	*18.44*	189.74	±	*3.78*
17	Timnodonic A (C20:5n3) - EPA	111.83	±	*11.03*	1593.45	±	*217.04*	548.24	±	*34.09*	501.54	±	*88.36*
18	Arachidonic A (C20:4n6)	3161.12	±	*327.83*	3632.39	±	*30.04*	4673.32	±	*288.23*	5649.10	±	*62.58*
19	DGLA (C20:3n6)	470.29	±	*54.50*	537.30	±	*131.79*	960.37	±	*61.37*	968.97	±	*19.58*
20	Eicosatrienoic A (C20:3n3) - ETE	7.17	±	*2.28*	17.62	±	*1.01*	17.45	±	*5.19*	8.86	±	*0.06*
21	Eicosadienoic A (C20:2n6)	27.25	±	*7.11*	24.39	±	*0.01*	34.12	±	*3.20*	48.83	±	*5.26*
22	Gondoic A (C20:1n9)	68.33	±	*25.80*	132.16	±	*0.51*	89.04	±	*0.17*	88.26	±	*8.62*
23	Arachidic A (C20:0)	19.67	±	*2.87*	46.64	±	*1.20*	49.35	±	*5.45*	101.14	±	*2.76*
24	Heneicosanoic A (C21:0)	3.20	±	*0.57*	2.99	±	*0.59*	2.84	±	*0.23*	2.38	±	*0.28*
25	Cervonic A (C22:6n3) - DHA	17.66	±	*16.10*	18.94	±	*0.86*	15.11	±	*12.69*	14.94	±	*1.90*
26	Docosadienoic A (C22:2n6)	4.62	±	*0.77*	7.44	±	*0.59*	3.92	±	*0.54*	4.49	±	*0.15*
27	Erucic A (C22:1n9)	15.25	±	*3.42*	19.65	±	*6.38*	12.60	±	*0.82*	11.77	±	*1.47*
28	Behenic A (C22:0)	235.18	±	*25.94*	175.41	±	*4.12*	175.85	±	*9.39*	408.49	±	*6.08*
29	Tricosanoic A (C23:0)	12.37	±	*1.85*	13.95	±	*0.89*	12.76	±	*0.55*	9.68	±	*0.29*
30	Nervonic A (C24:1n9)	271.86	±	*32.95*	164.90	±	*2.90*	47.60	±	*2.04*	13.20	±	*6.76*
31	Lignoceric A (C24:0)	157.42	±	*18.88*	135.24	±	*2.49*	165.00	±	*8.83*	196.14	±	*3.36*

Table S4: The average content of individual FAMEs in *Dryopteridaceae* fern samples.

	Species/Family<br>FAME (µg.g^{-1} DW)	*Dryopteris aemula*<br>*Dryopteridaceae*	*Dryopteris affinis*<br>*Dryopteridaceae*	*Dryopteris borreri*<br>*Dryopteridaceae*	*Dryopteris cambrensis*<br>*Dryopteridaceae*	*Dryopteris carthusiana*<br>*Dryopteridaceae*	*Dryopteris caucasica*<br>*Dryopteridaceae*	[H]*Dryopteris dilatata*<br>*Dryopteridaceae*
1	Caproic A (C6:0)	25.24 ± *1.57*	24.18 ± *0.90*	28.54 ± *3.80*	25.50 ± *4.27*	22.92 ± *0.27*	23.16 ± *1.29*	20.89 ± *1.11*
2	Caprylic A (C8:0)	3.29 ± *0.11*	3.13 ± *0.11*	3.52 ± *0.16*	5.18 ± *0.35*	2.92 ± *0.13*	2.78 ± *0.22*	2.52 ± *0.22*
3	Capric A (C10:0)	4.23 ± *0.01*	4.36 ± *0.06*	4.36 ± *0.17*	4.18 ± *0.32*	4.03 ± *0.18*	3.99 ± *0.27*	4.25 ± *0.19*
4	Lauric A (C12:0)	5.64 ± *0.55*	5.99 ± *0.30*	6.52 ± *0.46*	9.97 ± *0.18*	6.09 ± *0.24*	5.14 ± *0.61*	5.73 ± *0.33*
5	Myristoleic A (C14:1n5)	10.81 ± *0.24*	8.86 ± *0.13*	9.96 ± *0.09*	9.95 ± *0.59*	9.87 ± *0.63*	10.44 ± *0.49*	8.29 ± *0.53*
6	Myristic A (C14:0)	23.65 ± *2.31*	14.16 ± *0.06*	21.67 ± *0.92*	14.62 ± *1.15*	17.84 ± *0.61*	24.01 ± *0.29*	20.66 ± *0.88*
7	Pentadecanoic A (C15:0)	39.76 ± *1.13*	20.77 ± *1.26*	19.17 ± *0.41*	11.08 ± *9.24*	30.60 ± *0.88*	31.99 ± *0.69*	12.83 ± *11.64*
8	Palmitoleic A (C16:1n7)	351.00 ± *366.13*	388.09 ± *8.15*	465.11 ± *20.90*	427.92 ± *26.63*	461.43 ± *17.24*	797.43 ± *34.81*	629.86 ± *23.47*
9	Palmitic A (C16:0)	1770.20 ± *48.70*	1068.88 ± *67.29*	1391.46 ± *46.81*	994.11 ± *82.91*	1376.87 ± *80.31*	1792.02 ± *30.30*	1434.82 ± *87.67*
10	cis-10-Heptadecenoic A (C17:1n7)	15.38 ± *0.45*	18.38 ± *0.45*	17.34 ± *1.23*	20.58 ± *0.05*	19.28 ± *0.04*	18.54 ± *4.16*	17.97 ± *0.10*
11	Margaric A (C17:0)	46.68 ± *2.54*	40.73 ± *1.98*	37.99 ± *1.11*	40.35 ± *0.07*	56.40 ± *3.44*	52.50 ± *1.18*	47.00 ± *1.78*
12	gamma-Linolenic A (C18:3n6)	1618.36 ± *28.13*	1086.66 ± *34.65*	1745.97 ± *123.46*	975.57 ± *78.37*	1249.67 ± *61.34*	1770.06 ± *1.88*	1520.34 ± *46.79*
13	Linolenic A (C18:3n3)	3092.90 ± *50.35*	1576.83 ± *160.87*	1984.12 ± *110.11*	1568.83 ± *106.22*	2499.51 ± *106.93*	3416.47 ± *42.60*	2627.98 ± *38.05*
14	Linoleic A (C18:2n6c)	4744.69 ± *427.05*	2828.20 ± *19.91*	3226.28 ± *413.30*	3232.14 ± *232.06*	4016.83 ± *79.40*	5166.56 ± *54.30*	4046.01 ± *47.68*
15	Oleic A (C18:1n9c)	3651.67 ± *184.02*	2046.82 ± *20.44*	2754.73 ± *73.32*	2203.02 ± *127.30*	2762.80 ± *79.82*	4186.26 ± *72.02*	2795.75 ± *28.23*
16	Stearic A (C18:0)	101.13 ± *3.35*	69.88 ± *1.19*	100.92 ± *1.93*	64.19 ± *9.46*	117.07 ± *5.21*	142.54 ± *28.55*	108.21 ± *4.17*
17	Timnodonic A (C20:5n3) - EPA	292.94 ± *45.32*	381.43 ± *25.41*	492.38 ± *29.58*	369.32 ± *51.50*	490.65 ± *68.95*	722.25 ± *26.59*	298.10 ± *9.70*
18	Arachidonic A (C20:4n6)	3762.90 ± *50.61*	2560.64 ± *164.54*	3495.40 ± *350.94*	2514.86 ± *237.70*	3105.17 ± *53.33*	4380.75 ± *2.59*	3330.77 ± *153.57*
19	DGLA (C20:3n6)	1020.93 ± *12.72*	570.28 ± *16.31*	902.07 ± *72.19*	433.50 ± *22.20*	1260.01 ± *18.33*	957.32 ± *34.10*	1137.34 ± *21.97*
20	Eicosatrienoic A (C20:3n3) - ETE	9.65 ± *4.53*	18.52 ± *7.62*	14.07 ± *1.39*	11.33 ± *0.21*	19.29 ± *2.25*	16.72 ± *3.02*	13.98 ± *0.79*
21	Eicosadienoic A (C20:2n6)	41.28 ± *17.07*	30.90 ± *15.39*	27.47 ± *6.38*	22.41 ± *3.86*	44.62 ± *1.40*	42.85 ± *1.94*	42.55 ± *8.01*
22	Gondoic A (C20:1n9)	119.03 ± *2.17*	204.13 ± *0.20*	204.73 ± *8.87*	227.60 ± *14.46*	267.45 ± *0.02*	241.70 ± *23.93*	285.80 ± *9.14*
23	Arachidic A (C20:0)	104.81 ± *2.00*	80.35 ± *2.98*	94.17 ± *5.94*	91.66 ± *2.67*	215.31 ± *5.67*	108.46 ± *5.68*	110.11 ± *5.30*
24	Heneicosanoic A (C21:0)	4.88 ± *0.09*	8.17 ± *0.54*	7.73 ± *0.23*	8.20 ± *0.03*	17.88 ± *0.19*	8.92 ± *0.89*	7.22 ± *0.22*
25	Cervonic A (C22:6n3) - DHA	23.36 ± *0.16*	14.55 ± *11.07*	14.94 ± *11.45*	13.49 ± *8.81*	20.73 ± *19.62*	24.45 ± *2.51*	21.98 ± *0.56*
26	Docosadienoic A (C22:2n6)	4.91 ± *0.61*	6.32 ± *0.12*	8.02 ± *0.69*	7.19 ± *0.90*	7.35 ± *0.06*	7.23 ± *0.39*	8.88 ± *0.85*
27	Erucic A (C22:1n9)	6.50 ± *5.39*	8.03 ± *5.55*	9.85 ± *6.66*	10.11 ± *4.98*	18.05 ± *17.28*	6.27 ± *0.24*	30.98 ± *12.90*
28	Behenic A (C22:0)	170.35 ± *1.58*	203.78 ± *10.60*	208.03 ± *12.10*	162.30 ± *2.93*	221.14 ± *9.28*	221.33 ± *4.63*	208.86 ± *7.26*
29	Tricosanoic A (C23:0)	11.33 ± *0.39*	19.43 ± *25.14*	28.52 ± *1.79*	17.91 ± *22.40*	46.28 ± *0.71*	38.52 ± *2.48*	39.57 ± *0.11*
30	Nervonic A (C24:1n9)	224.99 ± *25.02*	99.13 ± *19.24*	104.70 ± *9.48*	69.62 ± *14.96*	131.51 ± *1.59*	151.85 ± *24.12*	150.76 ± *5.99*
31	Lignoceric A (C24:0)	197.33 ± *1.32*	150.96 ± *9.72*	148.39 ± *7.40*	135.15 ± *0.30*	138.66 ± *2.97*	158.86 ± *5.72*	157.05 ± *2.36*

Table S5: The average content of individual FAMEs in *Dryopteridaceae* fern samples.

	Species/Family<br>FAME ($\mu g.g^{-1}$ DW)	*Dryopteris expansa*<br>Dryopteridaceae	[H]*Dryopteris filix-mas*<br>Dryopteridaceae	*Dryopteris oreades*<br>Dryopteridaceae	*Dryopteris remota*<br>Dryopteridaceae	[H]*Polystichum aculeatum*<br>Dryopteridaceae	*Polystichum aculeatum*<br>Dryopteridaceae	*Polystichum setiferum*<br>Dryopteridaceae
1	Caproic A (C6:0)	24.20 ± *3.41*	22.28 ± *0.27*	22.85 ± *3.59*	23.83 ± *2.30*	22.90 ± *2.35*	23.70 ± *4.78*	19.93 ± *4.16*
2	Caprylic A (C8:0)	2.76 ± *0.08*	2.87 ± *0.21*	2.65 ± *0.04*	5.03 ± *0.08*	2.52 ± *0.15*	3.00 ± *0.06*	2.63 ± *0.17*
3	Capric A (C10:0)	4.23 ± *0.21*	3.93 ± *0.08*	3.84 ± *0.09*	5.55 ± *0.05*	3.67 ± *0.07*	3.92 ± *0.33*	3.84 ± *0.05*
4	Lauric A (C12:0)	4.99 ± *0.40*	5.86 ± *0.30*	5.15 ± *0.08*	8.35 ± *0.36*	5.04 ± *0.02*	5.60 ± *0.14*	4.97 ± *0.14*
5	Myristoleic A (C14:1n5)	10.37 ± *0.39*	9.35 ± *0.04*	9.41 ± *0.35*	9.48 ± *0.04*	8.41 ± *0.21*	9.12 ± *0.51*	10.54 ± *0.11*
6	Myristic A (C14:0)	24.87 ± *0.68*	22.39 ± *0.56*	17.37 ± *0.19*	20.51 ± *1.39*	30.47 ± *0.94*	25.05 ± *0.91*	35.13 ± *2.40*
7	Pentadecanoic A (C15:0)	27.67 ± *0.62*	25.45 ± *0.26*	11.36 ± *16.06*	21.69 ± *0.95*	17.31 ± *0.44*	27.52 ± *0.65*	18.15 ± *19.48*
8	Palmitoleic A (C16:1n7)	1342.11 ± *40.27*	428.61 ± *3.40*	458.82 ± *11.31*	56.32 ± *0.15*	393.19 ± *17.90*	391.74 ± *19.40*	475.47 ± *23.54*
9	Palmitic A (C16:0)	1914.39 ± *26.40*	1509.99 ± *87.73*	1310.25 ± *4.74*	1378.95 ± *43.28*	1783.41 ± *84.70*	1392.77 ± *63.79*	1581.31 ± *130.99*
10	cis-10-Heptadecenoic A (C17:1n7)	25.36 ± *2.34*	17.36 ± *0.32*	23.72 ± *0.58*	15.83 ± *0.30*	13.69 ± *0.86*	12.00 ± *0.14*	13.35 ± *1.06*
11	Margaric A (C17:0)	60.19 ± *7.18*	49.28 ± *0.44*	49.19 ± *1.97*	37.55 ± *1.66*	43.49 ± *1.11*	36.24 ± *2.73*	49.35 ± *2.01*
12	gamma-Linolenic A (C18:3n6)	2622.62 ± *37.60*	1614.35 ± *141.59*	1536.95 ± *35.13*	1581.63 ± *53.65*	1916.90 ± *87.61*	1077.47 ± *75.33*	1575.75 ± *167.97*
13	Linolenic A (C18:3n3)	4461.73 ± *22.81*	2961.72 ± *172.74*	2716.36 ± *114.10*	1963.78 ± *2.78*	3721.08 ± *155.08*	1965.81 ± *130.02*	2767.26 ± *150.70*
14	Linoleic A (C18:2n6c)	5008.30 ± *6.33*	4167.96 ± *170.78*	4628.72 ± *90.34*	4196.43 ± *769.50*	4299.44 ± *433.75*	4130.62 ± *704.55*	4064.88 ± *304.11*
15	Oleic A (C18:1n9c)	4826.76 ± *147.86*	3030.56 ± *126.34*	2975.79 ± *33.72*	2447.19 ± *38.83*	3424.16 ± *20.39*	2377.88 ± *221.31*	3097.68 ± *327.94*
16	Stearic A (C18:0)	147.68 ± *0.51*	119.35 ± *3.53*	91.97 ± *13.03*	92.80 ± *5.83*	107.20 ± *11.86*	134.84 ± *1.51*	88.07 ± *117.05*
17	Timnodonic A (C20:5n3) - EPA	962.06 ± *37.00*	322.78 ± *34.22*	206.09 ± *11.39*	545.17 ± *50.21*	330.11 ± *9.63*	211.04 ± *9.78*	315.10 ± *40.39*
18	Arachidonic A (C20:4n6)	4890.14 ± *176.99*	3301.80 ± *107.90*	3351.06 ± *86.70*	3322.04 ± *43.81*	4603.39 ± *90.13*	3743.65 ± *185.25*	4009.64 ± *366.05*
19	DGLA (C20:3n6)	1771.26 ± *59.35*	977.81 ± *73.03*	741.07 ± *43.94*	746.05 ± *0.84*	955.85 ± *85.02*	889.94 ± *55.85*	851.87 ± *136.70*
20	Eicosatrienoic A (C20:3n3) - ETE	32.28 ± *1.01*	15.69 ± *0.60*	16.31 ± *0.43*	15.65 ± *0.93*	8.33 ± *1.15*	14.04 ± *0.71*	14.70 ± *1.69*
21	Eicosadienoic A (C20:2n6)	54.67 ± *3.09*	72.79 ± *0.14*	33.41 ± *0.11*	83.61 ± *1.67*	68.35 ± *2.39*	58.78 ± *0.13*	65.75 ± *3.67*
22	Gondoic A (C20:1n9)	390.05 ± *66.61*	187.19 ± *9.81*	158.29 ± *14.37*	269.65 ± *30.03*	441.60 ± *9.85*	291.29 ± *73.73*	398.10 ± *5.50*
23	Arachidic A (C20:0)	224.89 ± *11.89*	90.75 ± *2.03*	89.24 ± *1.79*	92.77 ± *2.71*	170.12 ± *3.54*	223.39 ± *7.86*	234.17 ± *5.84*
24	Heneicosanoic A (C21:0)	11.93 ± *0.52*	8.51 ± *0.32*	7.51 ± *0.38*	7.87 ± *0.32*	7.48 ± *0.30*	9.35 ± *0.39*	10.39 ± *0.59*
25	Cervonic A (C22:6n3) - DHA	25.32 ± *0.11*	21.69 ± *1.68*	18.75 ± *0.81*	26.41 ± *0.50*	30.03 ± *0.12*	29.71 ± *2.09*	16.93 ± *14.47*
26	Docosadienoic A (C22:2n6)	14.00 ± *0.91*	6.49 ± *0.03*	5.10 ± *0.34*	6.51 ± *0.27*	6.76 ± *0.08*	6.12 ± *0.23*	10.62 ± *0.16*
27	Erucic A (C22:1n9)	24.49 ± *0.75*	22.20 ± *0.75*	7.03 ± *4.56*	9.11 ± *5.22*	11.58 ± *3.82*	9.53 ± *1.63*	16.69 ± *4.96*
28	Behenic A (C22:0)	304.12 ± *11.84*	172.17 ± *1.71*	183.30 ± *7.67*	190.18 ± *4.52*	361.05 ± *7.50*	369.25 ± *7.41*	466.73 ± *27.72*
29	Tricosanoic A (C23:0)	52.57 ± *4.28*	18.77 ± *23.95*	30.51 ± *1.48*	34.44 ± *1.34*	19.65 ± *0.62*	21.47 ± *0.39*	17.46 ± *0.62*
30	Nervonic A (C24:1n9)	313.15 ± *5.54*	96.88 ± *44.77*	132.15 ± *65.17*	123.95 ± *29.44*	6.43 ± *0.09*	46.77 ± *26.51*	122.83 ± *88.42*
31	Lignoceric A (C24:0)	178.00 ± *5.89*	137.43 ± *2.00*	140.21 ± *5.30*	132.10 ± *1.55*	174.79 ± *3.55*	146.93 ± *1.94*	123.28 ± *7.40*

Table S6: The average content of individual FAMEs in *Onocleaceae*, *Osmundaceae*, and *Polypodiaceae* fern samples.

	Species/Family<br>FAME (µg.g^{-1} DW)	[H]*Matteuccia struthiopteris*<br>*Onocleaceae*	*Onoclea sensibilis*<br>*Onocleaceae*	[H]*Osmunda regalis*<br>*Osmundaceae*	*Osmunda regalis*<br>*Osmundaceae*	[H]*Polypodium vulgare*<br>*Polypodiaceae*	*Polypodium vulgare*<br>*Polypodiaceae*
1	Caproic A (C6:0)	18.83 ± *0.27*	19.22 ± *2.86*	16.41 ± *0.38*	16.40 ± *1.42*	19.33 ± *0.45*	18.65 ± *7.75*
2	Caprylic A (C8:0)	2.86 ± *0.01*	0.00 ± *0.00*	0.00 ± *0.00*	0.00 ± *0.00*	2.68 ± *0.05*	2.87 ± *0.14*
3	Capric A (C10:0)	4.25 ± *0.24*	3.65 ± *0.47*	3.96 ± *0.01*	5.66 ± *0.13*	0.00 ± *0.00*	0.00 ± *0.00*
4	Lauric A (C12:0)	7.29 ± *0.65*	5.59 ± *0.65*	5.13 ± *0.17*	5.00 ± *0.00*	7.53 ± *1.29*	6.37 ± *0.04*
5	Myristoleic A (C14:1n5)	8.57 ± *1.24*	8.21 ± *1.09*	8.02 ± *0.11*	7.94 ± *0.25*	8.20 ± *0.12*	8.31 ± *0.49*
6	Myristic A (C14:0)	17.81 ± *2.08*	16.75 ± *0.90*	11.63 ± *0.20*	12.38 ± *0.16*	21.06 ± *1.96*	17.42 ± *0.07*
7	Pentadecanoic A (C15:0)	23.26 ± *2.78*	23.29 ± *1.58*	12.03 ± *0.28*	11.28 ± *0.66*	10.57 ± *0.38*	11.46 ± *0.69*
8	Palmitoleic A (C16:1n7)	914.86 ± *66.06*	394.63 ± *28.03*	254.59 ± *9.53*	190.15 ± *44.23*	447.69 ± *24.76*	402.57 ± *1.08*
9	Palmitic A (C16:0)	1934.22 ± *194.62*	1713.89 ± *111.30*	2043.22 ± *88.67*	2040.07 ± *1.77*	1944.43 ± *62.83*	1495.08 ± *41.73*
10	cis-10-Heptadecenoic A (C17:1n7)	18.39 ± *1.21*	13.47 ± *1.31*	12.66 ± *0.60*	10.99 ± *0.33*	15.35 ± *1.06*	14.53 ± *0.66*
11	Margaric A (C17:0)	41.30 ± *0.43*	48.91 ± *4.92*	46.83 ± *2.69*	36.60 ± *2.24*	43.97 ± *4.00*	32.22 ± *0.44*
12	gamma-Linolenic A (C18:3n6)	2151.42 ± *116.45*	1502.98 ± *145.41*	1097.34 ± *76.36*	1055.16 ± *34.78*	2572.32 ± *81.77*	1331.11 ± *54.25*
13	Linolenic A (C18:3n3)	3345.94 ± *356.72*	2434.59 ± *50.21*	4149.79 ± *260.63*	4118.78 ± *73.57*	3800.96 ± *236.30*	2341.05 ± *120.30*
14	Linoleic A (C18:2n6c)	4595.90 ± *347.18*	4868.95 ± *258.67*	3834.53 ± *236.71*	2791.62 ± *125.57*	7173.74 ± *141.34*	7250.33 ± *78.88*
15	Oleic A (C18:1n9c)	3023.33 ± *301.78*	2697.63 ± *264.53*	2784.61 ± *161.65*	2591.18 ± *1.20*	4568.90 ± *137.67*	3810.06 ± *23.40*
16	Stearic A (C18:0)	155.70 ± *15.78*	149.77 ± *0.11*	152.20 ± *31.37*	139.76 ± *46.21*	140.39 ± *5.32*	102.09 ± *4.03*
17	Timnodonic A (C20:5n3) - EPA	1828.21 ± *366.38*	397.97 ± *52.53*	999.17 ± *2.08*	1193.46 ± *232.08*	154.39 ± *49.24*	103.25 ± *4.99*
18	Arachidonic A (C20:4n6)	4301.51 ± *391.83*	3709.38 ± *192.89*	2678.83 ± *27.10*	2296.72 ± *174.62*	6257.19 ± *176.28*	5260.76 ± *136.99*
19	DGLA (C20:3n6)	1152.59 ± *15.60*	719.23 ± *48.03*	630.18 ± *77.91*	573.84 ± *52.57*	1688.38 ± *30.14*	1103.08 ± *53.52*
20	Eicosatrienoic A (C20:3n3) - ETE	42.76 ± *1.78*	15.64 ± *1.10*	30.80 ± *0.26*	25.70 ± *0.88*	26.91 ± *1.61*	21.30 ± *0.32*
21	Eicosadienoic A (C20:2n6)	47.25 ± *4.31*	56.12 ± *1.49*	35.84 ± *1.14*	55.41 ± *5.23*	77.34 ± *1.29*	81.18 ± *1.72*
22	Gondoic A (C20:1n9)	113.84 ± *6.15*	112.30 ± *2.00*	69.62 ± *8.48*	56.85 ± *4.00*	85.38 ± *38.18*	43.65 ± *0.89*
23	Arachidic A (C20:0)	46.00 ± *10.25*	48.23 ± *3.85*	41.29 ± *7.53*	12.81 ± *15.91*	166.94 ± *13.56*	97.41 ± *8.35*
24	Heneicosanoic A (C21:0)	2.97 ± *0.26*	2.40 ± *0.39*	3.59 ± *0.44*	2.35 ± *0.08*	3.96 ± *0.54*	3.21 ± *0.08*
25	Cervonic A (C22:6n3) - DHA	35.39 ± *5.93*	26.32 ± *2.32*	12.52 ± *0.29*	11.91 ± *0.15*	10.80 ± *4.21*	21.92 ± *20.06*
26	Docosadienoic A (C22:2n6)	8.63 ± *0.23*	2.41 ± *0.14*	2.85 ± *0.07*	1.83 ± *0.14*	3.13 ± *0.58*	2.47 ± *0.68*
27	Erucic A (C22:1n9)	16.61 ± *7.73*	5.95 ± *1.17*	4.20 ± *0.34*	3.41 ± *0.87*	3.78 ± *0.71*	3.10 ± *0.24*
28	Behenic A (C22:0)	177.74 ± *8.64*	137.88 ± *6.64*	203.90 ± *7.89*	171.45 ± *25.93*	233.97 ± *11.01*	177.03 ± *11.10*
29	Tricosanoic A (C23:0)	13.01 ± *0.82*	11.21 ± *0.48*	17.12 ± *0.25*	11.89 ± *0.42*	6.91 ± *0.42*	6.59 ± *0.51*
30	Nervonic A (C24:1n9)	22.38 ± *7.36*	39.20 ± *1.63*	19.24 ± *16.63*	8.80 ± *4.15*	68.65 ± *12.13*	9.49 ± *5.75*
31	Lignoceric A (C24:0)	142.94 ± *6.98*	166.55 ± *8.53*	99.00 ± *4.52*	85.80 ± *4.04*	123.56 ± *4.09*	112.40 ± *5.28*

Table S7: The average content of individual FAMEs in *Thelypteridaceae* fern samples and samples of reference vegetables.

	Species/Family<br>FAME (µg.g^{-1} DW)	*Lastrea limbosperma*<br>Thelypteridaceae			[W]*Lastrea limbosperma*<br>Thelypteridaceae			*Phegopteris connectilis*<br>Thelypteridaceae			*Thelypteris palustris*<br>Thelypteridaceae			*Eruca sativa*<br>Brassicaceae			*Spinacia oleracea*<br>Amaranthaceae		
1	Caproic A (C6:0)	22.11	±	*0.05*	20.40	±	*0.30*	16.62	±	*7.18*	25.53	±	*0.42*	14.00	±	*5.90*	14.37	±	*2.65*
2	Caprylic A (C8:0)	2.49	±	*0.15*	2.63	±	*0.14*	2.94	±	*0.19*	2.66	±	*0.07*	3.94	±	*0.00*	1.34	±	*1.90*
3	Capric A (C10:0)	4.29	±	*0.19*	4.39	±	*0.30*	3.92	±	*0.22*	3.91	±	*0.01*	4.72	±	*0.05*	3.62	±	*0.06*
4	Lauric A (C12:0)	5.48	±	*0.28*	5.90	±	*0.02*	6.08	±	*0.29*	7.41	±	*0.13*	20.21	±	*0.70*	8.28	±	*3.03*
5	Myristoleic A (C14:1n5)	9.25	±	*0.42*	9.30	±	*0.85*	8.78	±	*0.57*	9.62	±	*0.39*	7.59	±	*1.13*	7.59	±	*0.80*
6	Myristic A (C14:0)	28.03	±	*1.22*	23.96	±	*0.50*	31.94	±	*0.95*	61.53	±	*2.26*	21.51	±	*1.40*	19.07	±	*12.36*
7	Pentadecanoic A (C15:0)	19.97	±	*1.54*	25.67	±	*1.75*	18.18	±	*0.35*	16.08	±	*0.84*	17.33	±	*0.36*	26.25	±	*18.32*
8	Palmitoleic A (C16:1n7)	385.51	±	*7.75*	499.34	±	*34.66*	368.08	±	*14.65*	288.52	±	*337.40*	233.95	±	*24.06*	187.69	±	*120.78*
9	Palmitic A (C16:0)	1748.64	±	*20.21*	2072.57	±	*19.63*	2252.86	±	*50.75*	2015.77	±	*80.90*	1274.59	±	*36.99*	948.87	±	*684.67*
10	cis-10-Heptadecenoic A (C17:1n7)	15.04	±	*0.39*	16.86	±	*0.96*	13.64	±	*1.19*	17.62	±	*0.50*	18.36	±	*2.12*	9.88	±	*5.64*
11	Margaric A (C17:0)	34.33	±	*0.02*	55.88	±	*1.47*	46.33	±	*1.47*	40.84	±	*3.54*	107.42	±	*8.91*	28.06	±	*18.54*
12	gamma-Linolenic A (C18:3n6)	1503.95	±	*47.09*	1860.09	±	*29.16*	1390.92	±	*3.31*	1451.32	±	*112.91*	0.00	±	*0.00*	0.00	±	*0.00*
13	Linolenic A (C18:3n3)	2945.97	±	*30.17*	4753.61	±	*225.91*	3510.88	±	*81.19*	3274.88	±	*17.96*	7008.02	±	*372.76*	5771.87	±	*3635.94*
14	Linoleic A (C18:2n6c)	4153.07	±	*37.70*	4672.86	±	*47.90*	6553.86	±	*339.72*	5609.10	±	*388.22*	2658.57	±	*332.03*	1973.00	±	*1328.28*
15	Oleic A (C18:1n9c)	2741.55	±	*53.51*	3593.72	±	*160.56*	3782.75	±	*109.63*	3763.88	±	*88.66*	1803.48	±	*77.94*	1487.75	±	*1234.62*
16	Stearic A (C18:0)	123.28	±	*0.97*	160.42	±	*14.04*	350.07	±	*3.50*	144.53	±	*23.87*	235.63	±	*9.41*	92.56	±	*53.96*
17	Timnodonic A (C20:5n3) - EPA	501.09	±	*12.72*	726.05	±	*1.45*	514.72	±	*112.63*	1155.03	±	*24.66*	0.00	±	*0.00*	0.00	±	*0.00*
18	Arachidonic A (C20:4n6)	3755.09	±	*85.45*	3742.90	±	*83.03*	4434.28	±	*134.55*	3719.77	±	*230.97*	5.17	±	*0.24*	7.10	±	*0.84*
19	DGLA (C20:3n6)	640.94	±	*39.07*	575.70	±	*41.86*	638.49	±	*40.60*	724.66	±	*67.98*	0.00	±	*0.00*	0.00	±	*0.00*
20	Eicosatrienoic A (C20:3n3) - ETE	13.77	±	*0.27*	14.49	±	*0.48*	12.00	±	*0.88*	27.20	±	*1.13*	48.84	±	*0.53*	18.30	±	*10.40*
21	Eicosadienoic A (C20:2n6)	43.96	±	*1.30*	24.63	±	*1.86*	61.85	±	*5.70*	51.44	±	*12.93*	19.71	±	*0.38*	7.06	±	*1.65*
22	Gondoic A (C20:1n9)	89.92	±	*2.35*	124.74	±	*16.23*	99.33	±	*30.89*	111.18	±	*28.50*	42.97	±	*8.22*	66.14	±	*35.91*
23	Arachidic A (C20:0)	55.00	±	*18.56*	101.37	±	*1.27*	261.10	±	*10.80*	71.22	±	*22.26*	26.12	±	*0.26*	26.94	±	*18.83*
24	Heneicosanoic A (C21:0)	4.66	±	*0.20*	4.63	±	*0.11*	3.85	±	*0.25*	3.70	±	*0.24*	2.72	±	*0.17*	3.76	±	*1.73*
25	Cervonic A (C22:6n3) - DHA	12.65	±	*0.40*	17.88	±	*1.32*	14.60	±	*10.68*	15.40	±	*0.36*	19.33	±	*16.31*	7.41	±	*2.83*
26	Docosadienoic A (C22:2n6)	9.40	±	*0.18*	14.34	±	*1.91*	12.77	±	*1.46*	5.35	±	*0.14*	5.21	±	*0.92*	2.51	±	*0.88*
27	Erucic A (C22:1n9)	32.00	±	*0.82*	59.38	±	*4.31*	14.83	±	*3.10*	15.20	±	*2.85*	11.98	±	*2.17*	3.21	±	*2.15*
28	Behenic A (C22:0)	437.96	±	*9.32*	437.95	±	*23.43*	642.81	±	*29.26*	444.85	±	*51.78*	110.30	±	*3.45*	51.52	±	*36.53*
29	Tricosanoic A (C23:0)	13.77	±	*0.70*	11.34	±	*0.52*	15.43	±	*0.76*	12.94	±	*1.10*	25.22	±	*0.18*	7.66	±	*5.02*
30	Nervonic A (C24:1n9)	113.60	±	*18.28*	187.80	±	*50.37*	24.01	±	*0.06*	51.77	±	*3.31*	55.39	±	*15.52*	0.00	±	*0.00*
31	Lignoceric A (C24:0)	154.13	±	*5.93*	144.05	±	*5.26*	302.49	±	*12.48*	154.30	±	*20.52*	341.84	±	*1.07*	52.20	±	*37.38*
